# Quantitative Bone SPECT/CT in Diabetic Foot Osteomyelitis: Diagnostic Performance Within-Patient Lesion–Contralateral Separation and Associations with Inflammatory Burden

**DOI:** 10.3390/diagnostics15222907

**Published:** 2025-11-17

**Authors:** Hulya Peker Yalcin, Pınar Akkus Gunduz, Mehmet Samsum, Emel Colak Samsum, Aysenur Erol, Umut Mert Turan, Gulsah Gedikli Turgut, Aysun Yalci, Nihal Yesildag, Musa Fatih Yalcin, Nesibe Zeynep Eryavuz

**Affiliations:** 1 Department of Nuclear Medicine, Gulhane Education and Research Hospital, 06010 Ankara, Turkey; pakkus_@hotmail.com (P.A.G.);; 2 Department of Nuclear Medicine, Ankara University, 06560 Ankara, Turkey; 3 Department of Nuclear Medicine, Etlik Education and Research Hospital, 06170 Ankara, Turkey; mehmetsamsum@gmail.com; 4 Directorate General of Health Services, Turkish Ministry of Health, 06800 Ankara, Turkey; 5 Department of Infectious Diseases and Clinical Microbiology, Gulhane Educational and Research Hospital, University of Health Sciences, 06010 Ankara, Turkey

**Keywords:** diabetic foot osteomyelitis, bone SPECT/CT, quantitative SPECT, standardized uptake value (SUV), SUVmax, SUVmean, C-reactive protein (CRP), erythrocyte sedimentation rate (ESR)

## Abstract

**Objective**: We sought to assess the diagnostic performance of quantitative bone SPECT/CT standardized uptake values (SUVs) in diabetic foot osteomyelitis (DFO) and their associations with inflammatory biomarkers. **Methods**: We retrospectively reviewed 150 consecutive patients who underwent three-phase bone scintigraphy and foot SPECT/CT (November 2016–December 2024) for DFO before antibiotic treatment; 117 with complete imaging and laboratory data were analyzed. Lesion and contralateral SUVs (SUVmax, SUVmean) were compared. Receiver operating characteristic (ROC) curves were used to determine discrimination and optimal cut-offs (Youden index). Associations with biomarkers (C-reactive protein (CRP), erythrocyte sedimentation rate (ESR), CRP/albumin (ESR × CRP) and hematologic/coagulation indices including mean corpuscular hemoglobin (MCH) and activated partial thromboplastin time (aPTT) were evaluated using Spearman correlations. **Results**: Lesion uptake and contralateral uptake were SUVmax 10.94 ± 7.36 vs. 3.62 ± 1.70; SUVmean 4.38 ± 3.63 vs. 0.93 ± 0.50. Discrimination was excellent; SUVmax was AUC 0.921 (cut-off 4.47; sensitivity 0.93; specificity 0.75) and SUVmean was AUC 0.961 (cut-off 1.49; sensitivity 0.91; specificity 0.89). CRP and ESR showed weak but consistent positive correlations with SUVs (ρ ≈ 0.25–0.30). The ESR × CRP value correlated most strongly (e.g., with SUVmean ρ = 0.35), and CRP/albumin showed a modest positive association. MCH (ρ ≈ −0.20) and aPTT (ρ ≈ −0.37) were inversely related. **Conclusions**: Quantitative SPECT/CT provides excellent lesion–contralateral discrimination in DFO. SUVs—particularly SUVmean—track inflammatory burden, supporting their use as practical quantitative adjuncts to clinical and laboratory assessment. Study-specific cut-offs are promising but require local validation.

## 1. Introduction

Diabetic foot disease is among the most serious complications of diabetes mellitus (DM). Up to one-third of diabetic patients may suffer from diabetic foot disease during their life [1,2]. Problems affecting the foot in diabetic patients can be grouped under three main headings: acute infections of the foot, diabetic foot ulcers, and Charcot neuroarthropathy. Between 50% and 70% of non-traumatic lower extremity amputations are performed in patients with DM. It has been reported that 85% of patients undergoing major amputation had a pre-existing foot ulcer prior to the procedure. The recurrence rate of diabetic foot ulcers is approximately 20% within one year and increases to around 65% within 3–5 years. Diabetic foot is the leading cause of prolonged hospital stays among patients with diabetes. Within 3–5 years after the first amputation, more than 50% of patients require amputation of the contralateral limb. The development of a new foot ulcer in diabetic patients has been shown to increase the risk of mortality by approximately 2.5-fold. The 1-year mortality rate in patients with diabetic foot reaches 10%, while the 5-year mortality rate rises to 50–70% [3].

Osteomyelitis can develop when bacteria gain access to bone directly, spread to bone via the bloodstream from a distant infectious focus, or extend to bone from an adjacent soft-tissue infection through a contiguous route termed non-hematogenous spread [4].

Diagnosing osteomyelitis relies on a combination of clinical evaluation, laboratory testing, and imaging studies. In previous studies, it has been reported that inflammatory markers, including C-reactive protein (CRP), interleukin-6 (IL-6), tumor necrosis factor (TNF), glycosylated hemoglobin (HbA1c), and CRP/albumin ratio, play a role in the prognosis of diabetic foot [4,5,6,7]. A range of imaging modalities—plain radiography, magnetic resonance imaging (MRI), computed tomography (CT) and nuclear medicine procedures such as bone scintigraphy, gallium imaging, and 18F-fluorodeoxyglucose positron emission tomography/CT (18F-FDG PET/CT)—is essential for demonstrating both early and chronic manifestations of osteomyelitis. Imaging supports the diagnosis, helps to define disease extent and complications, and guides therapeutic planning [8].

Three-phase bone scintigraphy is a sensitive method for detecting osteomyelitis when the evaluated bone is not altered by other underlying disorders [9]. Bone scintigraphy is commonly performed with Technetium-99m (99mTc)-methylene diphosphonate (MDP), which attaches to bone hydroxyapatite. It reflects both regional perfusion and osteoblastic activity. In suspected osteomyelitis, a standard three-phase protocol is followed. First, a dynamic flow phase; second, early static images are taken during the blood pool/soft tissue phase; and third, delayed skeletal images are taken 2–4 h after tracer injection. In such studies, a combination of focal hyperperfusion, focal hyperemia, and increased delayed osseous uptake is considered typical for osteomyelitis [10]. The three-phase bone scan, combined with labeled leukocyte scintigraphy, demonstrates high diagnostic accuracy in differentiating osteomyelitis from osteoarthropathy, with sensitivity up to 100% and specificity of up to 83%. Labeled leukocyte scintigraphy (99mTc hexamethyl propylene-amine oxime (HMPAO) or Indium-111) accumulates at infection sites but not in healing bone, making it a valuable tool for monitoring treatment response. Despite its advantages of high sensitivity and specificity (approximately 80%), the technique is limited by low anatomical resolution, time-consuming acquisition, and reduced accuracy in diabetic osteoarthropathy or under antibiotic therapy [11,12,13].

Hybrid single photon emission computed tomography (SPECT) and high-resolution computed tomography (CT) (SPECT/CT) imaging are used for the precise anatomical localization of physiologic uptake to overcome the disadvantages of conventional imaging. With new developments in SPECT technology, the standardized uptake value (SUV), which is well known in PET imaging, is used for quantitative measurement in SPECT/CT [14].

In this study, we aimed to quantify within-patient lesion–contralateral separation using SPECT/CT-derived SUVs and to evaluate associations between SUV metrics and infection parameters.

## 2. Materials and Methods

In this retrospective study, a total of 150 diabetic foot patients who applied to the Nuclear Medicine Department of Gulhane Education and Research Hospital between November 2016 and December 2024, and who underwent 3-phase bone scan and SPECT/CT imaging, were analyzed. After the intravenous injection of 740 MBq 99mTc-MDP into an upper limb vein, flow and blood-pool images were acquired. At 3 h after injection, both planar and SPECT/CT images were obtained. All images were acquired on a Discovery NM/CT 670 (GE Healthcare, Chicago, IL, USA) using a low-energy, high-resolution (LEHR) collimator. The bone scintigraphy procedure guideline of the Society of Nuclear Medicine (version 3) was followed [15].

This study was conducted in accordance with the Declaration of Helsinki and was approved by the Institutional Ethics Committee of Gulhane Education and Research Hospital (protocol code 2025/210; date 9 October 2025). Because only de-identified retrospective data were analyzed, the requirement for informed consent was waived by the Committee. We used ChatGPT (GPT-5, a generative pre-trained transformer, to generate text, speech, and images in response to user prompts) to assist with figure preparation and help in the statistical analysis and interpretation.

### 2.1. SPECT/CT Acquisition and SUV Quantification

Imaging was performed on Discovery NM/CT 670 (GE Healthcare, Chicago, IL, USA) using LEHR collimators.

SPECT was acquired over 360° via 60 projections, applying 15 s/view in a noncircular orbit. The acquisition matrix was 128 × 128.

Low-dose CT (for attenuation correction and anatomical localization) was acquired with the following settings: 120 kVp, 50–100 mAs, pitch 1.25, slice thickness 2.5 mm and a field-of-view encompassing both feet.

### 2.2. Image Reconstruction and Corrections

SPECT data were reconstructed using OSEM with resolution recovery (8 iterations, 10 subsets) and CT-based attenuation correction. A Butterworth post-filter (10 mm FWHM) was applied.

System sensitivity was established by cross-calibration of the SPECT system with the dose calibrator using a uniform cylindrical phantom filled with 99mTc. All activities were decay-corrected to the start of the SPECT acquisition.

### 2.3. SUV Calculation

The injected activity (A_inj) was computed as the pre-injection syringe activity minus the residual post-injection, both decay-corrected to the injection time. Body weight (g) at imaging was also recorded.

Voxelwise activity concentration (C_tissue, kBq/mL) was converted to standardized uptake value (SUV_BW) as:

SUV_BW = C_tissue [kBq/mL]/(A_inj [kBq]/Body weight [g])

### 2.4. VOI Definition and Metrics

The 3D volume of interest (VOI) was placed by two nuclear medicine physicians who were blinded to each other and the clinical data. On fused SPECT/CT, a 3D VOI was manually drawn around the osteomyelitic lesion, guided by CT to exclude artifacts and adjacent high-uptake structures. From this lesion VOI, we automatically recorded the following:

- SUVmax—the maximum voxel within the VOI;
- SUVmean—the mean of all voxels within the VOI.

**Figure 1:**
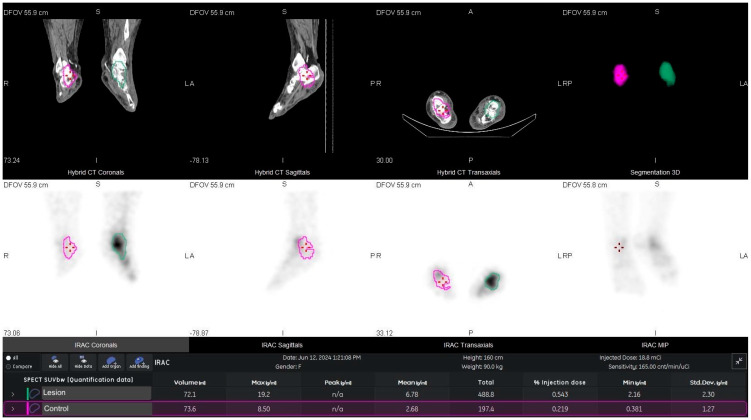
An example of SUVmax and SUVmean processing on 99mTc-MDP bone SPECT/CT with osteomyelitis on the left talus. (green line: osteomyelitis talus; purple line: contralateral talus).

An anatomically matched VOI of identical size was placed on the contralateral clinically unaffected foot to obtain control SUVmax and SUVmean. When minor motion or metal artifacts were present, VOIs were adjusted on CT to exclude contaminated voxels. No partial-volume correction was applied (Figure 1).

### 2.5. Laboratory Analysis

Laboratory data were collected retrospectively. The following inflammatory markers were included in the study: erythrocyte sedimentation rate (ESR), C-reactive protein (CRP), CRP/albumin ratio, as well as complete blood count and coagulation parameters, including mean corpuscular hemoglobin (MCH) and activated partial thromboplastin time (aPTT).

### 2.6. Statistical Analysis

Continuous variables were presented as median (IQR) or mean ± SD as appropriate, and categorical variables as *n* (%). Normality was assessed with the Shapiro–Wilk test and homogenity of variances with Levene’s test. For paired comparisons between the osteomyelitic foot and the contralateral side, SUVmax vs. control SUVmax and lesion SUVmean vs. control SUVmean, the Wilcoxon signed-rank test was used.

To evaluate the ability of SUVmax and SUVmean to distinguish the lesions from contralateral uptake, we constructed ROC curves, AUCs with 95% CIs (bootstrap) were reported, and the optimal cut-off was determined using the Youden Index (J = sensitivity + specificity − 1).

Associations between SUV metrics (continuous) and laboratory markers (e.g., CRP, ESR) were assessed with Spearman’s rank correlation (ρ). For group comparisons by ROC-derived SUV cut-offs (e.g., SUVmax ≥ 4.47), continuous outcomes were compared using Mann–Whitney U (or Welch’s t-test if normally distributed with unequal variances), and categorical outcomes (e.g., surgery, hyperbaric oxygen treatment, peripheral vascular disease) using Fisher’s exact test (or χ^2^ when expected counts ≥5). Where applicable, effect sizes were reported (odds ratios or risk differences with 95% CIs).

Analyses were performed using IBM SPSS Statistics version 26 (IBM Corp., Armonk, NY, USA) and Python 3.14.0 (Python Software Foundation, Wilmington, DL, USA) with scikit-learn and statsmodels.

## 3. Results

A total of 117 patients with diabetic foot were analyzed. The mean age was 61.37 ± 12.20 years and the mean BMI was 26.88 ± 3.29 kg/m^2^. Inflammatory markers were elevated, with a mean CRP of 39.77 ± 31.02 mg/L. Hematologic indices showed platelets 289.43 ± 106.69 × 10^3^/µL, lymphocytes 24.30 ± 9.31%, neutrophils 65.12 ± 9.98%, and WBC 8.82 ± 3.45 × 10^3^/µL. Glycemic control parameters were poor (fasting blood glucose 174.25 ± 85.63 mg/dL, HbA1c 8.68 ± 2.34%). Coagulation tests were largely within acceptable limits (PT 10.10 ± 1.99 s, aPTT 28.66 ± 3.51 s, INR 1.05 ± 0.18). Infection-related composite indices were also increased, with a CRP/albumin ratio of 8.70 ± 11.97, neutrophil-to-lymphocyte ratio 3.56 ± 3.06, and ESR × CRP 4228.14 ± 2246.19 (Table 1).

**Figure 2:**
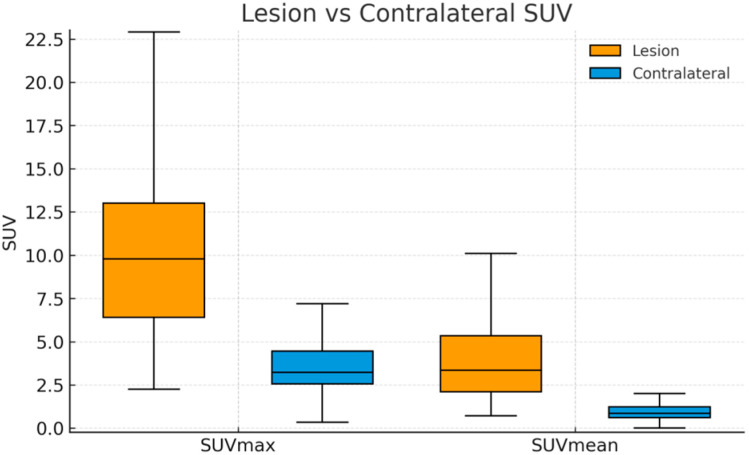
Lesion vs. contralateral SUV on 99mTc-MDP SPECT/CT. Colored boxplots (lesion = orange, contralateral = blue) show medians (center lines) and interquartile ranges (boxes); whiskers denote 1.5 × IQR. Lesion uptake exceeded contralateral for both SUVmax (9.79 [6.40–13.00] vs. 3.22 [2.56–4.46]) and SUVmean (3.35 [2.10–5.34] vs. 0.86 [0.61–1.23]); Wilcoxon signed-rank test, *n* = 117 paired measurements, both *p* < 0.001.

Metabolic activities of lesions were higher than the contralateral controls—lesion SUVmax 10.94 ± 7.36 and lesion SUVmean 4.38 ± 3.63—whereas contralateral SUVmax was 3.62 ± 1.70 and contralateral SUVmean 0.93 ± 0.50 (both *p* < 0.001) (Figure 1 and Figure 2).

Data from all 117 patients were analyzed by two nuclear medicine physicians. Interobserver reliability was assessed using intraclass correlation coefficients (ICC), and we found excellent agreement across all measures. For lesion SUVmax, the single-measures ICC was 0.989 (95% CI 0.983–0.993; F (116, 116) = 187.66, *p* < 0.001) and the average-measures ICC was 0.995 (0.992–0.997). For lesion SUVmean, ICC (single) was 0.979 (0.968–0.987) and ICC (average) 0.990 (0.984–0.993) (F = 99.59, *p* < 0.001). For contralateral SUVmax, ICC (single) was 0.950 (0.920–0.968) and ICC (average) 0.974 (0.958–0.984) (F = 40.59, *p* < 0.001). For contralateral SUVmean, ICC (single) was 0.914 (0.868–0.945) and ICC (average) 0.955 (0.929–0.971) (F = 22.18, *p* < 0.001). These results showed excellent observer-independent consistency, with very similar distributions between readers (Supplementary Table S1).

**Figure 3:**
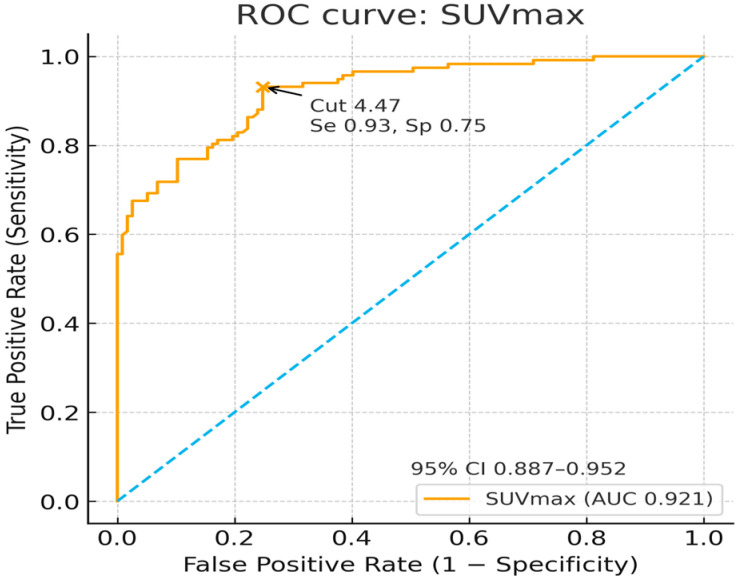
ROC curve discriminating the lesion from contralateral-side uptake using SUVmax. The curve (AUC = 0.921) showed the reported optimal operating point (cut-off = 4.47), shown with a marker and arrow (sensitivity = 0.93; specificity = 0.75).

**Figure 4:**
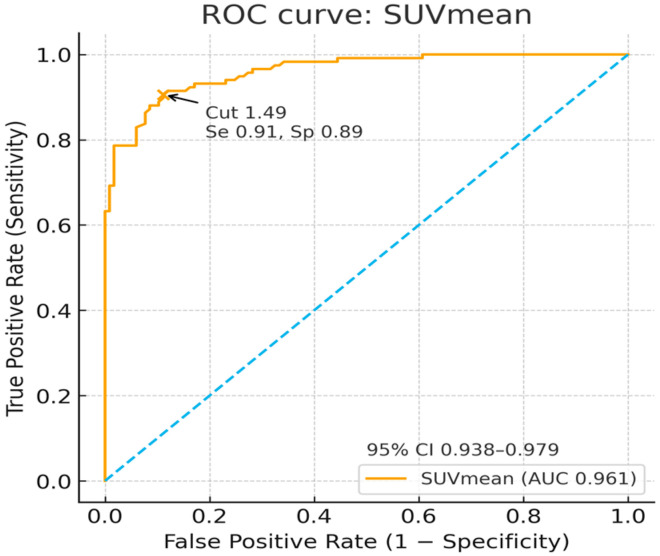
ROC curve discriminating the lesion from contralateral-side uptake using SUVmean. The curve (AUC = 0.961) showed the reported optimal operating point (cut-off = 1.49), highlighted with a marker and arrow (sensitivity = 0.91; specificity = 0.89).

Both measures discriminated lesion from contralateral uptake well. SUVmax had an AUC of 0.921 with an optimal cut-off of 4.47 (sensitivity 0.93, specificity 0.75). SUVmean had an AUC of 0.961 with a cut-off of 1.49 (sensitivity 0.91, specificity 0.89) (Figure 3 and Figure 4).

Lesion SUVs increased with systemic inflammation. For individual markers, CRP and ESR showed weak but consistent positive associations (Spearman ρ ≈ 0.25–0.30, all *p* ≤ 0.01). Composite indices strengthened this pattern; the ESR × CRP index showed the strongest association (e.g., with SUVmean ρ 0.35, *p* = 0.0002), and the CRP/albumin ratio showed a modest positive association (ρ 0.28, *p* = 0.007). Hematologic and coagulation measures were inversely related to lesion uptake. MCH showed a weak inverse correlation (e.g., SUVmean ρ −0.20, *p* = 0.039), and aPTT showed a moderate inverse correlation with both SUVs (e.g., SUVmean ρ −0.37, *p* = 0.0028) (Table 2).

A multiple linear regression was conducted to examine whether peripheral vascular disease, coronary artery disease, hypertension or chronic renal failure predict SUVs. The model was not significant for SUVs (SUVmax—F = 1.119, *p* = 0.356, R = 0.240, R^2^ = 0.058, adjusted R^2^ = 0.006; SUVmean—F = 0.953, *p* = 0.460, R = 0.222, R^2^ = 0.049, adjusted R^2^ = −0.002), and none of the predictors were significant on their own (all *p* > 0.05) (Supplementary Table S2).

In summary, lesion SUVmean and SUVmax were markedly elevated, provided excellent discrimination from the contralateral side with practical cut-offs, correlated positively with inflammatory burden (especially ESR × CRP and CRP/albumin), and showed inverse relationships with MCH and aPTT (Supplementary Figure S1).

## 4. Discussion

Diabetic foot is one of the most serious complications of diabetes mellitus and usually develops in the context of peripheral neuropathy, peripheral arterial disease, and superimposed infection. Diabetic foot ulcers are an important cause of morbidity and mortality, and account for most non-traumatic lower-extremity amputations [16]. The diagnosis is crucial for the prevention of amputation and the determination of antibiotic treatment duration [5,17,18,19,20]. Delayed diagnosis leads to late treatment initiation and allows the infection to spread [21,22].

Since the 1980s, nuclear medicine has proposed several strategies to separate true diabetic foot osteomyelitis from non-infectious neuropathic changes. Studies in which 99mTc-MDP bone scintigraphy was interpreted together with indium-111-labeled leukocyte imaging showed that the lesions accumulated radiotracer on both bone scans, and labeled leukocyte scintigraphy almost always showed osteomyelitis, confirmed by biopsy, whereas non-infectious neuro-osteoarthropathy, despite intense bone turnover, did not attract leukocytes [11,12,13]. Sensitivity in those series was approximately 100%, while specificity remained around 80% because of a small number of false-positive cases in which adjacent soft-tissue inflammation or complex postoperative change mimicked infection. A subsequent prospective study in diabetic adults confirmed that labeled leukocyte scintigraphy alone is highly sensitive, but that adding a three-phase bone study improves overall diagnostic performance, whereas gallium-67 contributes little in most cases [11]. Later, small prospective series included a delayed or “fourth” phase, and SPECT further increased diagnostic confidence, especially when structural foot deformity was present or MRI was equivocal [12].

The introduction of hybrid SPECT/CT systems addressed one of the persistent weaknesses of planar or SPECT-only studies, which was localization. This refers to the uncertainty of whether the focus of increased tracer uptake was actually located in bone tissue or in an adjacent inflamed soft tissue compartment, or in a degenerative/postoperative joint. By fusing functional and anatomical information, SPECT/CT reduced equivocal interpretations in deformed, postsurgical, or Charcot feet, and enabled a more accurate description of the spatial extent of the disease. More recently, quantitative SPECT/CT has been reported on, particularly with 99mTc-HMPAO-labeled leukocytes. Park et al. showed that SUVmax derived from WBC SPECT/CT was independently associated with subsequent amputation, and that a simple three-variable model (SUVmax, previous amputation, number of lesions) outperformed conventional binary SPECT/CT for prognostic stratification in diabetic foot infection [14]. These data suggest that the value of SPECT/CT in diabetic feet should not be limited to a yes/no decision on osteomyelitis, but can be extended to an objective measurement. However, leukocyte-labeled SPECT/CT is not available everywhere, requires experienced staff and more funds, and prolongs patient wait. It also may show reduced accuracy in patients who are already receiving systemic antibiotics or who have advanced diabetic osteoarthropathy [11,12,13]. In many nuclear medicine departments, what is consistently available and quickly performed is still 99mTc-MDP bone SPECT/CT. In such settings, it is clinically relevant to determine whether routinely acquired bone SPECT/CT—performed without labeled leukocytes—can be used quantitatively to support diagnosis or to stratify risk in patients with clinically suspected diabetic foot osteomyelitis.

Our cohort consisted of 117 patients who had a clinical diagnosis of diabetic foot infection/osteomyelitis established by the infectious diseases service, and who were treated accordingly. This represents real-life practice in many centers, where biopsy is reserved for selected or non-responding cases. We measured SUVmax and SUVmean from bone SPECT/CT in the clinically involved foot and compared them with measurements from the contralateral side. Both SUV parameters were clearly higher in the clinically infected limbs, and lesion-versus-contralateral discrimination was excellent, with AUCs of 0.921 for SUVmax and 0.961 for SUVmean; optimal cut-off values were 4.47 and 1.49, respectively. These results indicate that even bone SPECT/CT, which is traditionally regarded as a sensitive but not highly specific technique, contains useful quantitative information when acquired on modern hybrid systems and analyzed with SUV tools.

Our findings are also consistent with the literature on laboratory markers in DFO. Many studies have evaluated ESR, CRP, and more recently the CRP/albumin ratio as adjunctive tests for diagnosing diabetic foot osteomyelitis and for identifying patients at higher risk of amputation [6,7,23,24,25,26,27,28,29,30,31,32,33]. In general, these markers rise in the context of osteomyelitis; however, several authors have pointed out that diabetic patients often have ischemia, neuropathy, renal dysfunction or other comorbidities that decrease systemic inflammatory responses, so fever, leukocytosis, or high CRP may be absent even in extensive infection [28,29,30,31,32]. A meta-analysis related to ESR shows that ESR is a reliable serum inflammatory marker for the diagnosis of diabetic foot osteomyelitis. The results of these studies reveal that ESR has a sensitivity of 74–84% and a specificity of 56–75% for the diagnosis of diabetic foot osteomyelitis [33,34,35,36,37,38,39,40,41,42].

In studies that investigated serum CRP levels in diabetic foot osteomyelitis as a management tool and predictor for various diabetic diseases, the authors concluded that CRP levels are a good indicator for monitoring diabetic foot disease and are more effective and sensitive after appropriate therapy than the ESR [43,44]. In a systematic review, they evaluated the roles of ESR, CRP, platelets, and WBC in the diagnosis of diabetic foot ulcers according to the International Working Group on Diabetic Foot and Perfusion, Extent (size), Depth (tissue loss), Infection, Sensation classification system. From the analysis, they concluded that CRP is the ideal marker for distinguishing grade 2 diabetic foot ulcers from non-infected diabetic foot ulcers, especially in smaller communities lacking access to advanced imaging modalities [45].

In their study, Coye et al. evaluated the effectiveness of CRP/albumin, ESR, and CRP in differentiating bone and soft tissue infections in patients with diabetes, and found that CRP/albumin ratios provided results comparable to those of ESR and CRP alone [46]. In our study, in correlation with previous studies, we also found that SUVmax and SUVmean were modestly correlated with the ratio of CRP to albumin.

Although ESR and CRP are routinely used as cheap, well-established, and reliable markers of inflammation, both biomarkers demonstrate low mean sensitivity and specificity (ESR mean sensitivity—81.6%, mean specificity,—79.0%; CRP mean sensitivity—84.5%, mean specificity—81.3%) [47,48]. To address these inconveniences in predicting infections in non-orthopedic settings, the ratio between ESR and CRP (ECR) is used, yielding more successful results than using these parameters separately [49,50,51,52]. Moreover, in our study, as stated in previous studies, SUVmax and SUVmean values have a higher correlation with ESRxCRP value.

In the literature, conflicting results exist for specific parameters, such as the influence of hypertension, fasting blood glucose level, and HbA1c level on the prognosis of diabetic foot osteomyelitis; therefore, further clinical data and evidence are needed [43,53,54,55,56,57]. Our data show non-statistically significant correlations with fasting blood glucose level and HbA1c.

In the study by Radisic Biljak et al., the authors examined the relationship between increased factor VIII levels and shortened aPTT, suggesting that chronic inflammation may promote a hypercoagulable state through the upregulation of factor VIII [58]. In agreement with this finding, Esmon provided a detailed description of the reciprocal interplay between inflammation and coagulation. Pro-inflammatory cytokines such as IL-1β, IL-6, and TNF-α have been shown to stimulate tissue factor expression on monocytes, enhance the synthesis of fibrinogen and factor VIII, and at the same time suppress natural anticoagulant mechanisms, including antithrombin and the protein C system. Furthermore, inflammatory mediators downregulate thrombomodulin and the endothelial protein C receptor, thereby limiting the activation of protein C, while neutrophil-derived enzymes and oxidants degrade thrombomodulin and further diminish anticoagulant capacity [59,60]. These processes collectively increase thrombin generation and shift the hemostatic balance toward a procoagulant profile, which may result in shorter aPTT values even in the absence of an overt bleeding disorder.

Likewise, chronic inflammation can interfere with erythropoiesis through cytokine-driven hepcidin elevation, iron sequestration, and the inhibition of erythroid precursor activity, ultimately producing anemia of chronic disease. This state of functional iron deficiency leads to reduced mean corpuscular hemoglobin (MCH) and hemoglobin concentration despite adequate total iron stores [61,62].

In our study, the inverse associations observed between lesion SUVs and both aPTT and MCH are in line with these mechanisms. Taken together, our results suggest that higher SUV uptake, as an indicator of local and systemic inflammatory activity, is accompanied by inflammation-related alterations in coagulation and erythropoietic pathways, consistent with inflammation-induced coagulopathy.

## 5. Limitations

The study has several limitations that must be considered before these cut-off values are generalized. First, bone biopsy with histopathology and culture—considered the diagnostic reference standard—was not available. In some of the previous studies, clinical diagnosis by an experienced infectious diseases physician was considered as an acceptable reference. Second, all extremities belonged to patients with diabetes mellitus; our contralateral feet therefore do not represent healthy controls. Subclinical neuropathic, ischemic, or inflammatory changes may also have been present in the uninvolved limb and could have partially reduced the apparent contrast between sides. Third, limitations in SUV quantification are due to the acquisition method, particularly in small or heterogeneous foci. Noise and reconstruction artifacts can lead to an overestimation of SUVmax, whereas SUVmean may be underestimated because of partial volume effects. The applied Butterworth post-filter may further contribute to partial volume loss, as smoothing spreads counts into surrounding voxels—especially for small lesions—resulting in an underestimation of SUVmax. Fourth, the study was conducted at a single center; inter-institutional reproducibility was not assessed. These factors mean that the numerical thresholds we report (SUVmax 4.47; SUVmean 1.49) should be regarded as exploratory and should be re-evaluated in larger, preferably biopsy-verified, prospective series.

## 6. Future Directions

Prospective, multicenter studies with quantitative SPECT/CT, MRI and microbiology should validate site-specific cut-offs for SUVmax and SUVmean, test adjusted logistic models that incorporate vascular status, ulcer characteristics, and laboratory benchmark SPECT/CT SUVs, and explore threshold-free risk scores combining SUVmean, CRP, and clinical variables to guide biopsy, revascularization, hyperbaric oxygen treatment, and surgical timing.

## 7. Conclusions

Despite limitations, this study offers some practical contributions. First, quantitative bone SPECT/CT appears to be feasible in routine diabetic foot populations in which biopsy is not systematically performed. Second, SUVmean showed slightly better discrimination than SUVmax. This is plausible in DFO where infection is often fragmented, cortical, adjacent to an ulcer or sinus tract, or located close to prosthetic material. Under such circumstances, SUVmax is vulnerable to noise, small misregistrations, or a single very hot voxel, whereas SUVmean summarizes the uptake of the entire lesion volume and is therefore a more stable descriptor of total inflammatory/osteoblastic activity. We therefore suggest that quantitative SPECT/CT reports in DFO should provide both SUVmax and SUVmean, but SUVmean should be prioritized for longitudinal comparisons and for research analyses. Finally, these data fit within a broader trend in diabetic foot care: decisions are increasingly based on combinations of clinical, laboratory, vascular, and imaging findings, rather than on any single test. Our results indicate that, where leukocyte-labeled studies cannot be performed, quantitative bone SPECT/CT can supply objective, side-to-side information that aligns with systemic inflammation and that may help to identify a subgroup of patients with a truly “metabolically active” foot lesion. Future studies should test these observations in cohorts with bone biopsy confirmation, should standardize VOI definition and reconstruction across vendors, and should examine whether adding SUVmean or SUVmax from bone SPECT/CT to clinical laboratory models (e.g., CRP, CRP/albumin, ulcer depth, prior amputation) improves the prediction of amputation or treatment failure in a statistically and clinically meaningful way.

## Figures and Tables

**Table 1:** Demographic, clinical, and laboratory data of patients with diabetic foot.

	Patients (*n* = 117)
Age (years)	61.37 ± 12.20
BMI (kg/m^2^)	26.88 ± 3.29
**SPECT/CT Findings**	
SUVmax Lesion	10.94 ± 7.36
SUVmean Lesion	4.38 ± 3.63
SUVmax Control	3.62 ± 1.70
SUVmean Control	0.93 ± 0.50
**Laboratory Data**	
CRP (mg/L)	39.77 ± 31.02
Platelets (%)	289.43 ± 106.69
Lymphocytes (%)	24.30 ± 9.31
Neutrophil (%)	65.12 ± 9.98
WBC (%)	8.82 ± 3.45
MCH (pg)	27.70 ± 2.27
FBG (mg/dL)	174.25 ± 85.63
HbA1c (%)	8.68 ± 2.34
Albumin (g/dL)	4.36 ± 4.01
PT (s)	10.10 ± 1.99
aPTT (s)	28.66 ± 3.51
INR	1.05 ± 0.18
**Infection variables**	
CRP/Albumin	8.70 ± 11.97
Neutrophil/Lymphocytes	3.56 ± 3.06
ESR × CRP	4228.14 ± 2246.19

WBC—white blood cells, MCH—mean corpuscular hemoglobin, HbA1c—glycosylated hemoglobin, FBG—fasting blood glucose, INR—international normalized ratio, CRP—C-reactive protein, BMI—body mass index, ESR—erythrocyte sedimentation rate, PT—prothrombin time, aPTT—activated partial thromboplastin time.

**Table 2:** Correlations between SUV metrics and inflammatory/hematologic markers.

Comparison	Spearman ρ	95% CI	*p*-Value
SUVmax vs. CRP	0.25	0.06–0.42	0.008
SUVmax vs. ESR	0.28	0.09–0.45	0.004
SUVmean vs. CRP	0.29	0.08–0.11	0.003
SUVmean vs. ESR	0.3	0.09–0.12	0.002
SUVmean vs. ESR × CRP	0.35	0.20–0.51	0.0002
SUVmean vs. CRP/Albumin	0.28	0.07–0.46	0.007
SUVmean vs. MCH	−0.2	−0.37–0.07	0.039
SUVmean vs. aPTT	−0.37	−0.58–0.12	0.0028

MCH—mean corpuscular hemoglobin, CRP—C-reactive protein, ESR—erythrocyte sedimentation rate, aPTT—activated partial thromboplastin time.

## Data Availability

The original contributions presented in this study are included in the article. Further inquiries can be directed to the corresponding author.

## Supplementary Materials

The following supporting information can be downloaded at: https://www.mdpi.com/article/10.3390/diagnostics15222907/s1, Table S1: İnter-observer reliability test results of the two nuclear medicine physicians showed an excellent observer-independent consistency, with very similar distributions between readers; Table S2: Multiple linear regression used to examine peripheral vascular disease, coronary artery disease, hypertension and chronic renal failure to predict SUVs; Figure S1: STROBE-style flow diagram of our study.
