# Supplementary material for: Quantitative Bone SPECT/CT in Diabetic Foot Osteomyelitis: Diagnostic Performance Within-Patient Lesion–Contralateral Separation and Associations with Inflammatory Burden
Source: Diagnostics (Basel). 2025 Nov 17;15(22):2907. doi: 10.3390/diagnostics15222907 (PMC12651408; doi:10.3390/diagnostics15222907)

	Physician 1 (Mean ± SD)	Physician 2 (Mean ± SD)	ICC Single (95% CI)	ICC Average (95% CI)	F	p
**Lesion SUVmax**	10.54 ± 6.26	10.62 ± 6.21	0.989 (0.983–0.993)	0.995 (0.992–0.997)	187.66	<0.001
**Lesion SUVmean**	4.21 ± 3.12	4.34 ± 3.12	0.979 (0.968–0.987)	0.990 (0.984–0.993)	99.59	<0.001
**Contralateral SUVmax**	3.60 ± 1.50	3.48 ± 1.55	0.950 (0.920–0.968)	0.974 (0.958–0.984)	40.59	<0.001
**Contralateral SUVmean**	0.95 ± 0.53	0.93 ± 0.50	0.914 (0.868–0.945)	0.955 (0.929–0.971)	22.18	<0.001

SD: Standart deviation, ICC intraclass correlation coefficient

Table S1: İnter-observer reability test results of the two Nuclear Medicine physicians showed an excellent observer-independent consistency**,** with very similar distributions between readers

		r	Lower CI	Upper CI	p_raw	p FDR
SUVmax lesion	Hypertension	-0.1908	-0.3599	-0.0096	0.0393	0.1645
SUVmax lesion	Coronary artery disease	-0.1756	-0.3461	0.0061	0.0582	0.1979
SUVmax lesion	Peripheral vascular disease	-0.1504	-0.3231	0.0320	0.1055	0.2701
SUVmax lesion	Chronic kidney disease	0.1453	-0.0372	0.3184	0.1181	0.284
SUVmean lesion	Hypertension	-0.1623	-0.3340	0.0198	0.0804	0.2211
SUVmean lesion	Coronary artery disease	-0.1725	-0.3432	0.0094	0.063	0.1979
SUVmean lesion	Peripheral vascular disease	-0.1500	-0.3228	0.0324	0.1064	0.2701
SUVmean lesion	Chronic kidney disease	0.0572	-0.1257	0.2363	0.5403	0.6857

Table S2: A multiple linear regression to examine peripheral vascular disase, coronary artery disease, hypertension, chronic renal failure predict SUVs'. The model was not significant, for SUVs' (for SUVmax; F =1.119, p=0.356, R=0.240; R^2=0.058; adjusted R^2=.006),(for SUVmean (F = 0.953, *p* = 0.460, R =0 .222, R^2 =0 .049, adjusted R^2 = −0.002).

Figure S1: STROBE-style flow diagram of our study

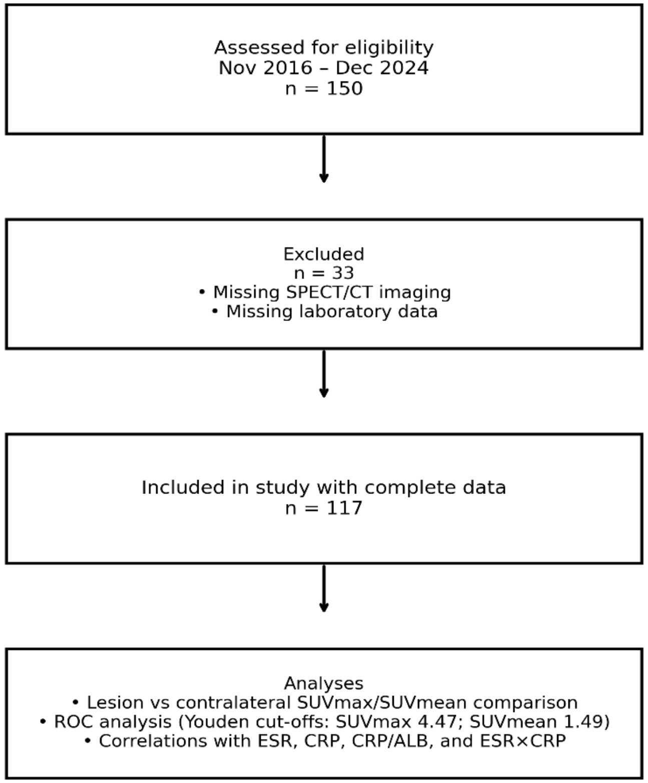

Supplement: Supplementary file 1 [file diagnostics-15-02907-s001.zip › diagnostics-3957597-supplementary.pdf]
